# Self-Focused and Other-Focused Health Concerns as Predictors of the Uptake of Corona Contact Tracing Apps: Empirical Study

**DOI:** 10.2196/29268

**Published:** 2021-08-10

**Authors:** Fenne große Deters, Tabea Meier, Anne Milek, Andrea B Horn

**Affiliations:** 1 Weizenbaum Institute for the Networked Society Berlin Germany; 2 University of Potsdam Potsdam Germany; 3 University Research Priority Program University of Zurich Zurich Switzerland; 4 University of Münster Münster Germany; 5 Center for Gerontology University of Zurich Zurich Switzerland

**Keywords:** COVID-19, corona contact tracing app, digital proximity tracing, preventive behavior, health concern, prosocial motivation, public health, risk perception, eHealth, Corona-Warn-App, SwissCovid, contact tracing app, contact tracing

## Abstract

**Background:**

Corona contact tracing apps are a novel and promising measure to reduce the spread of COVID-19. They can help to balance the need to maintain normal life and economic activities as much as possible while still avoiding exponentially growing case numbers. However, a majority of citizens need to be willing to install such an app for it to be effective. Hence, knowledge about drivers for app uptake is crucial.

**Objective:**

This study aimed to add to our understanding of underlying psychological factors motivating app uptake. More specifically, we investigated the role of concern for one’s own health and concern to unknowingly infect others.

**Methods:**

A two-wave survey with 346 German-speaking participants from Switzerland and Germany was conducted. We measured the uptake of two decentralized contact tracing apps officially launched by governments (Corona-Warn-App, Germany; SwissCovid, Switzerland), as well as concerns regarding COVID-19 and control variables.

**Results:**

Controlling for demographic variables and general attitudes toward the government and the pandemic, logistic regression analysis showed a significant effect of self-focused concerns (odds ratio [OR] 1.64, *P*=.002). Meanwhile, concern of unknowingly infecting others did not contribute significantly to the prediction of app uptake over and above concern for one’s own health (OR 1.01, *P*=.92). Longitudinal analyses replicated this pattern and showed no support for the possibility that app uptake provokes changes in levels of concern. Testing for a curvilinear relationship, there was no evidence that “too much” concern leads to defensive reactions and reduces app uptake.

**Conclusions:**

As one of the first studies to assess the installation of already launched corona tracing apps, this study extends our knowledge of the motivational landscape of app uptake. Based on this, practical implications for communication strategies and app design are discussed.

## Introduction

### Background

In March 2020, the World Health Organization declared the outbreak of the novel coronavirus SARS-CoV-2 a pandemic [1], and worldwide governments took radical measures to reduce the rate of transmission [2]. Most of these measures like quarantining potentially infected individuals, reducing social contact, and wearing face masks have been used for centuries to limit the spread of contagious diseases [3,4]. Technological advances in the 21st century have added contact tracing apps (CTAs) to our toolbox. Unsurprisingly, many governments are interested in CTAs as an additional measure to keep the pandemic under control [5]. In December 2020, a database by the MIT Technology Review listed 48 countries that have or are in the process of developing CTAs [6]. In the overwhelming majority of cases, the usage of such an app is voluntary or is planned to be voluntary. Simulation studies suggest that 56% of a population must use the app for an effect on the overall development of case numbers [7]. To reach this goal, it is important to understand what motivates citizens to adopt CTAs [8]. CTAs are a new phenomenon and, so far, early research mostly assessed app uptake intention before CTAs were launched [9]. Moreover, only a few studies have focused on underlying psychological factors motivating app uptake [8,10-12]. This study aimed to add to this line of research by investigating the role of self-focused and other-focused concerns regarding COVID-19 as so far understudied predictors of the adoption of two already launched CTAs.

### COVID-19 and Contact Tracing

An important measure to reduce the transmission of COVID-19 is contact tracing [13]. Individuals who have been physically close to an infected person receive a warning by health officials that they might have caught the disease and, even if they are not (yet) showing any symptoms, might be a spreader of the virus [14]. Warned individuals should then self-isolate and get tested [15,16]. By breaking the chain of infection, contact tracing can reduce the spread of the virus and help to contain the pandemic [13].

However, manual contact tracing has three problems. First, with exponentially rising numbers of cases, health officials are quickly overwhelmed with the workload. This results in a slower pace or even complete failure of informing individuals. Second, infected individuals might not recall all encounters they had in the critical time period. Finally, in case of contact with strangers like on public transportation, they might simply lack information on individuals with whom they have spent time in close proximity [5].

CTAs can mitigate these problems. Automatic contact tracing reduces the time between a positive test result and sending a warning to contacts of the infected person [17]. This is crucial for breaking the chain of infection, especially since presymptomatic transmission of COVID-19 appears to be common [14]. Automatic tracing via apps can also be scaled up more easily [18] and CTAs do not rely on the information on contacts provided by the infected individual [15].

### Corona CTAs

CTAs are apps for smartphones that keep track of other smartphones that have been in close physical proximity, and in case a smartphone owner tests positive for COVID-19, they allow sending a warning to these other smartphones. CTAs can be broadly classified into three different categories based on the role of the central server and the types of data that are stored by it (centralized, decentralized, and hybrid) [5]. These different architectures have implications for privacy protection and data security. Hence, they might influence the outcome of benefit-risk analyses and might determine the willingness to adopt a technology [19-21].

This study focuses on two decentralized CTAs officially launched by governments (Corona-Warn-App, Germany; SwissCovid, Switzerland). Decentralized CTAs prioritize privacy protection. The users’ identities are unknown to both other users and the central server [5]. Both the German and Swiss CTAs rely on the Google/Apple API, use Bluetooth Low Energy Technology to communicate with other smartphones, and can be downloaded for free [22-24]. SwissCovid (Switzerland) was released on June 25, 2020, to the general public and was downloaded 3 million times until March 2021 [25]. Corona-Warn-App (Germany) was released on June 16, 2020, and was downloaded 26.5 million times until March 2021 [26]. With around 6.8 million smartphone users in Switzerland [27] and around 66.5 million smartphone users in Germany [28], only 44% and 40%, respectively, of all potential users, in the most optimistic scenario, might have already adopted the apps. This highlights the need to understand drivers to increase app uptake.

### Benefits of App Uptake for Oneself and For Others as Potential Motivators

The benefits of using CTAs for oneself are not straightforward. Other apps in the health care sector like fitness or therapy apps promise to address a personal health problem or lower a risk for the user [12]. However, CTAs do not protect the user from COVID-19. These apps only warn individuals *retrospectively* that they have been exposed to the virus after a contact person has tested positive [5]. The value provided by CTAs results in a social dilemma–like situation. On the one hand, a wide acceptance of CTAs benefits all because this could lower the spread of the virus in the population. On the other hand, each individual has costs (time, inconvenience, etc) in case of installing the app, but no direct and clear health benefits or risk reduction [29].

Users only gain information on the likelihood of currently being infected. However, while not the focus of CTAs, individuals might use this information to evaluate their past behaviors in order to protect their health in the future. If warnings occur, they can review situations they have been in during the critical time period from which the warning resulted and try to avoid these.

Another direct benefit for a user is the decreased risk of unknowingly infecting others. Even if individuals feel healthy, they might be carriers of COVID-19 and spread the virus with potentially severe consequences for others [14]. Whether visiting a family member or spending time in a restaurant, a multitude of situations involve the risk of not only getting infected, but also accidentally harming others and having to face the guilt [30]. Particularly for individuals who expect to experience no dramatic symptoms if they catch COVID-19, reducing the risk of unknowingly infecting others might be a major selling point for CTAs. In short, app uptake might be motivated by being concerned about others rather than by being concerned about one’s own health. Our study examines the role of self-focused and other-focused concerns about COVID-19 for app uptake.

### Concerns as Drivers of Health-Compliant Behaviors

From the broader literature on preventive behaviors in the health context, it is evident that risk perception influences the likelihood of taking protective measures. The Protection Motivation Theory [31,32] and the Health Belief Model [33,34], two prominent theories in the area of health behavior promotion, suggest that risk perception can be a strong driver for individuals to engage in preventive behaviors [35]. Several studies have found positive associations between risk perception and health-compliant behaviors like social distancing and hand washing during the current pandemic [36,37] and previous disease outbreaks [35]. In other words, adoption of preventive behaviors is more likely if individuals think that they might be individually affected by the health problem. Risk perception can be approached from a cognitive or an emotional perspective. The emotional component of risk perception is characterized by worrying or being concerned about a threat. In the literature, these emotional facets of risk perceptions are seen as important predictors of favorable health behaviors [30]. In a recent study testing several theoretically derived variables as predictors of the intention to install a CTA, feeling anxious that oneself or a close other contracts COVID-19 emerged as a significant predictor [11]. There are some contradicting results in the literature, however, which have been interpreted by some as reflecting the effect of fear control instead of danger control responses. Too strong emotional reactions to a risk might overwhelm the individual, particularly if measures to reduce the threat are perceived as only moderately effective, and lead to defensive reactions and the rejection of preventive behaviors [38]. This boomerang effects can be tested by including curvilinear associations in the analysis [38-40].

Overall, it can be argued that certain characteristics of the situation during a novel pandemic likely impede with cognitive assessment of risks and render emotional aspects more important. The situation is quickly evolving, high levels of uncertainty exist due to often preliminary or contradictory information, and there is an acute threat [30]. Research conducted during the influenza A/H1N1 pandemic showed that emotional aspects of risk perception predicted protective behaviors over and above cognitive aspects [41] and emerged as stronger and more consistent predictors [42]. Hence, this study focused on concerns as the emotional dimension of risk perception.

#### Concern for One’s Own Health and App Uptake

Feeling at risk might motivate individuals to engage in preventive behaviors. However, according to the Protection Motivation Theory [31,32] and the Health Belief Model [33,34], this should only be the case if a certain behavior is perceived as effective in reducing the threat [35]. As outlined above, CTAs are not designed to decrease the risk of COVID-19 for an individual user. This raises the question whether concern for one’s own health is linked to app installation.

Several studies have reported that many participants (32% to 84%) list “protect my own health” among their reasons for installing such a CTA [18,43-47]. The association between perceived personal risk and intended app uptake was only assessed by a few studies and with mixed results. In some studies, the cognitive dimension of risk perception, namely the severity and susceptibility of infection, did not significantly predict the intention to install a CTA [8,10], whereas other studies found positive associations between personal risk and willingness to adopt a CTA [16,18,48-50]. These inconclusive results and the lack of studies assessing the emotional dimension instead of the cognitive dimension of risk perception warrant further research.

Another open question is whether very high levels of concern are linked to lower instead of higher app uptake. Too intense concerns might be overwhelming and may paralyze individuals or provoke defensive reactions, and hence, hinder them to take preventive actions [30]. Such a curvilinear relationship between concern and health-related behaviors has not yet been tested in respect to CTAs.

#### Concern for Others and App Uptake

Infectious diseases like COVID-19 that are transmitted by close contact to others inherently have a social dimension [30]. As social animals, we worry about not only our own welfare but also the welfare of others [51]. In the context of COVID-19, research has shown that consideration of others plays a role in the adoption of preventative health behaviors like wearing face masks, curtailing social contact, being willing to get vaccinated, or refraining from concealing potential COVID-19 symptoms [52-54]. Given that CTAs are intended to reduce the risk of the spread of the virus and hence protect others, it seems reasonable to assume that concern to infect others is associated with app uptake.

Previous research has shown that potential users are aware that CTAs protect others, and for a majority (52%-68%), this is an important reason to install the app [18,43,45-47]. In a discrete choice experiment on preferences for different app attributes, positive societal effects had a large impact on the probability to install a CTA [50]. Participants preferred app configurations that promised the prevention of deaths and long-term financial problems of households. However, as the authors note themselves, despite the collective framing, participants might have had their own well-being in mind, when evaluating the different app configurations. Another experimental study directly compared different motivations for app installation [12]. Participants either learned that by using the app they could (1) make an important contribution to their own health, (2) make an important contribution to the health of the population, or (3) both. Participants’ intentions to install a CTA were the highest if advertised with a procommunal effect and the lowest if only advertised with a personal health benefit. Apart from the three benefit appeals, two privacy designs and two convenience designs were manipulated in a full factorial experimental design. The authors do not report any tests for interaction effects. This warrants a cautious interpretation because the main effects could be qualified by an interaction. Moreover, the study assessed intention to use instead of actual app uptake. Ample research has demonstrated that intentions do not necessarily translate to actual behavior [55]. Accordingly, two studies on CTAs reported a gap between the intention and the actual installation of the app [45,56]. Moreover, due to the clear prosocial framing in the experiment, social desirability bias might have been an important factor influencing its outcome. Rejecting the installation of an app that benefits oneself is certainly more socially acceptable than being indifferent to the death and suffering of others [57].

Taken together, it remains an open question how self-focused and other-focused concerns relate to app installation. This might be relevant information to tailor promotion strategies, particularly in light of the unusual incentive structure CTAs offer.

### Our Study

This study explored the association between self-focused and other-focused concerns and app uptake. Given the novelty of the situation, this research is exploratory and assesses the following research questions: (1) What is the role of concern for one’s own health and concern to infect others in predicting the installation of a CTA? (2) Are very high levels of concern in comparison to moderate levels linked to a lower instead of higher likelihood of using a CTA?

In order to obtain robust results, we included potential confounders in our analyses. Apart from demographics like gender and age [18,49], disagreement with the classification of COVID-19 as a serious health crisis and the evaluation of the government’s pandemic policies are of interest. Both have been linked to the likelihood of taking preventive measures during pandemics [18,48,58-60]. Moreover, individuals holding the believe that an infectious disease has been hyped up can be expected to be less concerned about its effects. Moreover, feeling that the government is handling the health crisis satisfactorily might be linked to concerns. A positive evaluation of the government’s pandemic policies might be reassuring and associated with lower levels of risk perception [61]. On the contrary, perceiving the drastic measures like lockdowns issued by the governments of Switzerland and Germany [62] as justified might be associated with higher corona-related concerns. Moreover, a study in Singapore showed that higher confidence that the government would be able to handle the pandemic predicted installation of a CTA [63]. Hence, we included these variables in our study to control for confounding effects.

As another robustness check, we tested whether the association between app uptake and concerns depends on the timing of measuring concerns. Two opposing processes might influence concurrent associations between concerns and app uptake. First, engaging in recommended health behaviors might reduce a perceived health threat [64]. Hence, assessing concerns and app uptake only at the same point in time might result in finding a negative association or no association even if concerns actually motivated installation. Second, instead of feeling protected by the app, some participants expected increased feelings of anxiety as a result of app usage [18,47]. App users potentially receive warnings that they have been in close proximity to a COVID-19 carrier, and the icon of the app on the smartphone might act as a reminder of a threat. Hence, associations between concerns and app uptake in cross-sectional studies might reflect the reverse temporal order, that is, app usage preceding high levels of concern. Therefore, we included longitudinal analyses predicting app uptake with concerns measured before app release.

So far, research on drivers for the adoption of CTAs is scarce. Exploring the motivational landscape might enhance communication strategies and create ideas for features in the app. This study examines the interplay between self-focused and other-focused concerns in predicting app uptake while considering potential confounders, considering temporal aspects, and testing a curvilinear association. In contrast to most previous research, we focused on the emotional aspect of risk perception and assessed not just the intention to install a CTA but predict the actual (self-reported) installation of two already launched apps in real life.

## Methods

### Sample and Procedures

Data were collected online on the survey-platform Qualtrics [65] in two waves (T1: April 16-June 27, 2020; T2: July 1-September 18, 2020) as part of a multinational initiative studying how our everyday lives are affected by COVID-19 (Ashokkumar & Pennebaker, The Pandemic Project: Exploring the Social Dynamics of COVID-19, unpublished, 2020). The subsample for this study consists of German-speaking participants from Switzerland and Germany, which are two countries that had launched a CTA shortly before the data collection of the second wave took place. Reported COVID-19 case numbers and deaths remained low in both countries after the introduction of the CTA and the beginning of the data collection until its end. Similarly, measures and policies to control COVID-19 were comparable in both countries and remained largely unchanged during this time [66-68]. The link to the study was distributed by all authors via social media, mailing lists, newsletters, the Senior Citizens’ University of Zurich study participant pools, and websites of universities, as well as the website of the popular science magazine “Psychologie heute.” Fifteen participants were recruited via the participant recruitment platform Prolific. Prolific participants were eligible if German was their first language, they were living in Germany or Switzerland, and they were at least 50 years old. The goal here was to diversify the sample by recruiting more middle-aged and older participants.

With their permission, participants of the first wave received an invitation for the second wave, which included an ID to link the data of both waves. Additionally, new participants were recruited for the second wave via the aforementioned recruitment channels. The app-related questions were only included in the second wave after the launch of the CTAs. The final sample for the main analyses (N=346) consisted of all participants who answered those questions in wave 2. The sample for the additional longitudinal analyses included a subsample (N=270) of participants who had also participated in the first wave. Prior to data analyses, all data were anonymized.

Table 1 provides an overview of relevant demographic variables. In comparison to the general population in Switzerland and Germany, the sample included more participants who identified as female, had a higher education, and indicated their political orientation as left [69-74].

After opening the link to the study, general information about the topic of the study was provided. Participants learned that their participation was voluntary, and received information about data protection and their rights. After giving informed consent, participants provided demographic information. Next, participants were prompted to write about their thoughts and feelings related to the coronavirus outbreak. Then, participants proceeded to answer questions related to COVID-19 and their daily life during the pandemic (waves 1 and 2) and questions about the installation of a CTA (wave 2). In the last step, participants received feedback (eg, how preoccupied they are with the pandemic) and advice based on psychological research on how to cope with the situation. Prolific participants were compensated with £2.5 (US $1.4), and psychology students could receive course credit if eligible.

**Table 1:** Demographics (N=346).

Demographic variable		Value, n (%) or mean
**Subsample**		
	Germany	114 (32.9%)
	Switzerland	232 (67.0%)
**Gender**		
	Male	82 (23.7%)
	Female	262 (75.7%)
	Diverse/no answer	2 (0.6%)
**Age (years)**		
	18-29	70 (20.2%)
	30-39	74 (21.4%)
	40-49	66 (19.1%)
	50-59	32 (9.2%)
	60-69	46 (13.3%)
	70-79	50 (14.5%)
	80-89	8 (2.3%)
Mean age (years)		46.65
**Highest education**		
	Higher education (bachelor’s degree, master’s degree, PhD)	232 (67.1%)
	Higher education entrance qualification	59 (17.1%)
	Vocational training	45 (13.0%)
	Lower to intermediate secondary education	8 (2.3%)
	Other/no degree	2 (0.6%)
**Political orientation**		
	Extremely or somewhat left wing	219 (63.3%)
	In the middle	63 (18.2%)
	Extremely or somewhat right wing	32 (9.2%)
	I do not want to tell	32 (9.2%)

### Measures

#### Predictors

Table 2 and Table 3 provide information on the measures included as nondemographic predictors. All constructs were measured with a single item on a 5-point Likert scale ranging from “Not at all” (score 1) to “To a great deal” (score 5). “Concern self” and “Concern others” were measured in both wave 1 (T1) and wave 2 (T2).

**Table 2:** Nondemographic predictors.

Variable	Item English	Item German	Score, mean (SD)
1. Concern self (T2)	To what degree are you worried about getting COVID-19.	In welchem Ausmaß machen Sie sich Sorgen, selbst an COVID-19 zu erkranken.	2.15 (0.92)
2. Concern self (T1)	To what degree are you worried about getting COVID-19.	In welchem Ausmaß machen Sie sich Sorgen, selbst an COVID-19 zu erkranken.	2.10 (1.00)
3. Concern others (T2)	To what degree are you worried about unknowingly infecting others.	In welchem Ausmaß machen Sie sich Sorgen, unwissentlich Andere zu infizieren.	2.93 (1.22)
4. Concern others (T1)	To what degree are you worried about unknowingly infecting others.	In welchem Ausmaß machen Sie sich Sorgen, unwissentlich Andere zu infizieren.	3.11 (1.20)
5. Satisfaction with the government (T2)	I am satisfied with how my government has handled the COVID crisis.	Ich bin damit zufrieden wie meine Regierung mit der COVID-19 Krise umgegangen ist.	3.70 (0.86)
6. Not perceiving COVID-19 as a health crisis (T2)	To what degree do you feel that people are making too big a deal about COVID-19.	Inwieweit sind Sie der Meinung, dass die Leute eine zu große Sache aus COVID-19 machen.	2.11 (1.11)

**Table 3:** Correlations of nondemographic predictors.

Variable		Concern self (T2)	Concern self (T1)	Concern others (T2)	Concern others (T1)	Satisfaction with the government (T2)	Not perceiving COVID-19 as a health crisis (T2)
**Concern self (T2)**							
	*r*	1	0.62	0.32	0.24	0.18	−0.34
	*P* value	–^a^	<.001	<.001	<.001	.001	<.001
**Concern self (T1)**							
	*r*	0.62	1	0.15	0.23	0.16	−0.28
	*P* value	<.001	–	.01	<.001	.01	<.001
**Concern others (T2)**							
	*r*	0.32	0.15	1	0.66	0.13	−0.29
	*P* value	<.001	.01	–	<.001	.02	<.001
**Concern others (T1)**							
	*r*	0.24	0.23	0.66	1	0.13	−0.34
	*P* value	<.001	<.001	<.001	–	.03	<.001
**Satisfaction with the government (T2)**							
	*r*	0.18	0.16	0.13	0.13	1	−0.38
	*P* value	.001	.01	.02	.03	–	<.001
**Not perceiving COVID-19 as a health crisis (T2)**							
	*r*	−0.34	−0.28	−0.29	−0.34	−0.38	1
	*P* value	<.001	<.001	<.001	<.001	<.001	–

^a^N/A: not applicable.

#### Outcome

The outcome was measured in wave 2 after the release of the CTAs in Switzerland and Germany. Participants were asked whether they have installed a CTA like “SwissCovid” or “Corona-Warn-App” with the following response options: “Yes;” “Yes, but already uninstalled;” “No;” and “No, but I will likely do it.” The majority of the participants (202/346, 58.4%) had installed a CTA. The remaining participants indicated that they had not installed the app (111/346, 32.1%), had not yet installed it but will likely do so (25/346, 7.2%), or had already uninstalled it (8/346, 2.3%).

### Analytical Strategy

We recoded answers on app installation into a binary variable. If participants answered with “yes,” their answer was recoded as “app currently installed,” and all other answers (“no;” “not yet installed it but will likely do so;” and “yes, but already uninstalled it”) were recoded as “app currently not installed.” Using the R package “stats” [75], we ran logistic regression models to predict app installation. First, two separate models for the predictors “Concern self (T2)” and “Concern others (T2)” were calculated to show their univariate effect (models M1a and M1b). Next, both variables were included in the same model simultaneously to assess their unique contribution (model M2). Finally, we explored how the results changed when controlling for “Satisfaction with the government” and “Not perceiving COVID-19 as a serious health crisis.” Moreover, we added nationality, age, gender, highest education, and political orientation as control variables (model M3). To ease interpretation, we transformed the logistic regression coefficients to odds ratios (ORs), that is, the expected change in the odds of having the app installed if the predictor increases by one unit or changes from the reference category to another category in the case of categorical variables [76].

At each step, likelihood ratio tests showed a significantly improved model fit of the model with more predictors in comparison to the previous model with fewer predictors [77]. Starting with a model with the control variables and then adding “Concern self” and “Concern others” also resulted in an improved model fit. Because of the focus of the paper, we began with the two concern variables. Participants with missing values for any of the variables were excluded, which resulted in a sample size of 340 for these analyses. To get unbiased regression coefficients, an events per variable ratio of 1:10 or higher has been recommended for logistic regression [78,79]. With 140 participants in the category with fewer answers (“app currently not installed”), the events per variable ratio in the final model was 1:10.

#### Model Evaluation

As recommended for logistic regression models, we compared observed and predicted values to evaluate the fit of our model [80]. We calculated the area under the receiver operating characteristic curve (AUC, also known as c statistic). The AUC is the proportion of randomly drawn pairs of participants with different observed outcomes for which the model correctly predicts a higher probability of app uptake for the participant who has the app versus for the participant who does not. It ranges from 0.5 to 1. A value of 0.5 indicates that the model is not better than completely random assignment, while a value of 1 shows perfect performance [77].

#### Longitudinal Analyses

Concerns were measured at T1 as well before the release of the CTAs in Germany and Switzerland. This allowed us to test whether the association between app uptake and concerns depends on the timing of measuring concerns. We repeated all analyses with the concern variables measured at T1 (models M4a-M6). Not all participants had completed the questionnaire at T1, and analyses could only be performed with a sample of 270. The proportion of app users remained the same in this sample. Moreover, we assessed whether changes in concerns were associated with app uptake. We ran linear regression models predicting “Concern self (T2)” with app uptake while controlling for “Concern self (T1)” and our control variables. We repeated this analysis for “Concern others (T2).”

#### Curvilinear Association

To test whether too high levels of concern lead to defensive reactions, a curvilinear association of concerns with app uptake was tested. We mean-centered “Concern self (T2),” calculated the squared term for “Concern self (T2),” and added it to the final model. We repeated the same steps for “Concern others (T2)” (models M7 and M8).

## Results

### Predicting App Uptake With Concerns

All results are displayed in Table 4. Both “Concern self (T2)” and “Concern others (T2)” showed significant univariate associations with app uptake. Namely, the higher the concern, the higher the likelihood of currently using a CTA (models M1a and M1b). However, if both concerns were included as predictors, only “Concern self (T2)” significantly predicted app installation with an OR of 1.73. “Concern others (T2)” did not contribute significantly to the prediction of app uptake over and above worry for one’s own health (model M2).

Controlling for demographic variables and attitudes toward the government and the pandemic in general reduced the effect of “Concern self (T2)” slightly (model M3). Holding all other variables constant, with a one unit increase in “Concern self (T2),” the odds of currently having an app installed were 1.64 times higher. As expected, the less individuals perceived COVID-19 as a serious health crisis, the lower was the probability of app installation. Higher “Satisfaction with the government” was significantly associated with increased app uptake. Three demographic variables emerged as significant predictors. First, all else equal, participants in the Swiss subsample were more likely to have the app installed than participants in the German subsample. It should be noted that because the Swiss and German subsamples were not representative of the respective populations, the significant effect of the subsample in our study should not be interpreted as differences in app uptake between the two countries in general. Moreover, access to study populations and available recruiting strategies differed slightly between the authors in Switzerland and Germany. The variable was included in the model to assess the influence of concerns independent of these differences. Second, with older age, the likelihood of app uptake decreased. Finally, in comparison with the reference category of individuals with a degree in higher education, individuals with a “Higher education entrance qualification” were less likely to have a CTA installed.

**Table 4:** Logistic regression models predicting app uptake.

Model			*b*	SE	*P* value	95% CI		OR^a^
						Lower	Upper	
**M1a**								
	Concern self (T2)		0.58	0.13	<.001	0.32	0.84	1.78
**M1b**								
	Concern others (T2)		0.19	0.09	.03	0.02	0.37	1.21
**M2**								
	Concern self (T2)		0.55	0.14	<.001	0.28	0.83	1.73
	Concern others (T2)		0.07	0.10	.46	−0.11	0.26	1.07
**M3**								
	Concern self (T2)		0.50	0.16	.002	0.19	0.81	1.64
	Concern others (T2)		0.01	0.11	.92	−0.21	0.24	1.01
	Satisfaction with the government		0.45	0.17	.007	0.13	0.79	1.57
	Not perceiving COVID-19 as a health crisis		−0.35	0.13	.007	−0.61	−0.10	0.70
	Subsample Switzerland		0.65	0.29	.02	0.09	1.22	1.91
	Gender female		−0.54	0.33	.10	−1.19	0.09	0.58
	Age		−0.03	0.01	.002	−0.04	−0.01	0.98
	**Education (reference: higher education)**							
		Higher education entrance qualification	−1.14	0.36	<.001	−1.85	−0.44	0.32
		Vocational training	0.39	0.40	.33	−0.38	1.21	1.48
		Lower to intermediate secondary education	0.15	0.85	.86	−1.47	1.96	1.17
		Other/no degree	−0.64	1.49	.67	−3.96	2.69	0.53
	**Political orientation (reference: in the middle)**							
		Extremely or somewhat left wing	0.05	0.34	.87	−0.62	0.71	1.06
		Extremely or somewhat right wing	−0.80	0.51	.12	−1.82	0.20	0.45
		I do not want to tell	−0.56	0.52	.28	−1.59	0.45	0.57

^a^OR: odds ratio.

#### Model Evaluation

For the final model with all variables, the AUC was 0.74. Hence, the model correctly predicted for 74% of all nonapp user/app user pairs a higher probability for a participant who indeed has the app installed.

### Longitudinal Analyses

Repeating the analyses with concerns measured at T1 before app release, the pattern of results remained the same (Multimedia Appendix 1). “Concern self (T1)” significantly predicted higher app uptake with a similar OR (OR_T1_ 1.81, OR_T2_ 1.64), whereas “Concern others (T1)” had no significant effect. The AUC was 0.79. Accordingly, the linear regression models predicting change in concerns revealed no significant effect of app uptake on change in “Concern self” (*b*=0.14, *t_255_*=1.44, *P*=.15) or change in “Concern others” (*b*=−0.00, *t_255_*=−0.00, *P*>.99) (Multimedia Appendix 2 and Multimedia Appendix 3).

### Curvilinear Association

In the last step (models M7 and M8; Multimedia Appendix 4 and Multimedia Appendix 5), we tested for a curvilinear association between concerns and app uptake. For the quadratic term of “Concern self (T2),” no significant effect emerged (*b*=−0.09, SE=0.12, *P*=.44, 95% CI −0.32 to 0.15, OR 0.91). Similarly, the quadratic term in the model for “Concern others (T2)” was also nonsignificant (*b*=−0.08, SE=0.08, *P*=.32, 95% CI −0.25 to 0.08, OR 0.92). The likelihood ratio test confirmed that including curvilinear effects of concerns did not significantly improve model fit.

## Discussion

### Summary and Discussion of the Findings

As one of the first studies to assess the installation of already launched CTAs, this study contributes to our understanding of different motivations for app uptake. The results showed that concern for one’s own health predicts the installation of a decentralized CTA (OR 1.64, *P*=.002). Meanwhile, the concern to infect others did not contribute significantly to the prediction of app uptake over and above self-focused concern (OR 1.01, *P*=.92). In other words, individuals who had higher levels of worry to infect themselves with COVID-19 had downloaded a CTA with a higher probability, while being more or less concerned about unknowingly infecting others did not make a difference. This pattern held while controlling for demographics and attitudes toward the government and the pandemic. Longitudinal analyses replicated these results, thus supporting their robustness, and indicated that app uptake was not linked to changes in concerns. No evidence was found that “too much” concern leads to defensive reactions and reduces app uptake.

CTAs provide the following prominent and direct benefit to each individual user: more information on their likelihood to be currently infected with COVID-19. A warning by the app can prevent an individual from infecting someone else if appropriate measures like self-isolation are taken. Hence, it is surprising that in comparison to being concerned about one’s own health, being concerned about unknowingly infecting others does not significantly predict app uptake, particularly as participants in our sample reported on average more concern for others than for themselves and the variability of this variable was higher. Previous studies have shown that individuals are aware of the potential of the app to protect others [18,43,45-47]. However, perhaps concern for others is just not a sufficiently strong driver to motivate individuals to overcome the hassles and potential disadvantages of app installation. Research assessing both self-focused and other-focused concerns in the context of transmission-mitigating behaviors is scarce. Guillon and Kergall [48] found a similar pattern in their study. Participants who expected high individual health consequences were significantly more likely to be willing to use a CTA in the future. However, expected health impacts of COVID-19 in their country of residence did not predict the intention to use such an app.

Our result that self-focused concern is associated with app uptake is in line with studies that assessed the role of personal threat in predicting the intention to install a CTA [16,18,49,50] and other COVID-related health behaviors [36,37]. Given that CTAs are not designed to decrease the risk of a COVID-19 infection for an individual user, these results are nevertheless unexpected. It is possible that individuals are not aware of the limitations of CTAs in that regard. Maybe they falsely assume that using the app will protect them from infection. In a study on the Australian CTA, the majority of the participants thought that the app would detect when COVID-19 carriers are near them [81]. In a qualitative study conducted in Germany and Switzerland before the release of the CTAs, the same misconception was expressed by several participants [82]. Some of the official communication around the assessed CTAs might foster such a misunderstanding. For example, in a promotion video for the German CTA, the app promises “Ich beschütze Dich und sage Bescheid, wenn es Ernst wird” (I will protect you and I will let you know when it gets serious) [83].

Even if individuals are aware of how CTAs work, they might perceive the apps as at least somewhat effective in reducing the threat. As already outlined in the Introduction section, individuals might use the information provided by the app to evaluate their past behavior and avoid certain situations in the future.

It is also possible that the association between self-focused concern and app uptake is not due to an expected health benefit, but rather due to a higher need for uncertainty reduction, which is linked to higher anxiety [84]. Maybe getting more information on the current likelihood of being infected, as imperfect as the information may be, might be perceived as positive. Individuals might hope that elevated levels of concern due to situations with contact to many others like grocery shopping will be mitigated by monitoring potential warnings by the app. Another explanation is that individuals with high concerns regarding their personal health are just in general more motivated to follow any measures that promise to control the crisis, despite no direct personal health benefit.

Starting to use a CTA might provoke emotional responses. However, our longitudinal analyses do not support the possibility that app uptake is linked to changes in levels of concern. We found no evidence for increases in concern due to an increased awareness of the threat through app usage or decreases due to having adopted a recommended measure. At the very least, such effects did not appear uniformly across participants. This is in line with previous studies, which also did not report a significant change in concerns depending on app uptake [56,85]. During times with higher numbers of COVID-19 cases and hence a higher prevalence of warnings by the app, this might change.

### Practical Implications

While more research is needed to solidify, explain, and test the generalizability of our results, they suggest some practical implications. First, as self-focused concern could be the underlying motivation to install a CTA, implementing features that provide more information to users about the time of the exposure to COVID-19 might increase app uptake. This information might be perceived as helping individual risk management. With more detailed information on the timing of exposure, app users might be able to learn which situations hold a high risk of exposure and hence can try to avoid them in the future (eg, grocery shopping on a Saturday versus during the week). Moreover, they might remember more details about the specific situation (eg, whether it was outside or inside or whether everybody was wearing masks) and therefore feel enabled to better evaluate the actual risk of an infection. Decentralized CTAs already store timestamps. These data are necessary to calculate whether an encounter was long enough to pose a substantial risk and for how long a warning needs to be displayed until the app user’s potential period of infectiousness ends [5]. Naturally, there is always a trade-off between data protection and the benefits that come with collecting and sharing more data [21]. If warnings include detailed information on the timing of encountering a COVID-19 carrier, the identity of the infected person might get exposed. This could be mitigated if the granularity of the time information adapts in a way that, for example, at least five encounters with noninfected users have taken place during the same time window as well.

Second, app promotion could build on the fact that if individuals are worried about a COVID-19 infection, it is in their own best interest that everybody around them, like family, friends, or colleagues, uses a CTA. Highlighting this in official campaigns and framing messages in a way that directly encourages individuals to ask their close contacts to install the app might be an effective way to increase app uptake. After all, it is probably easier to ignore an official advertisement campaign for prosocial behavior than to ignore grandma, friends, or colleagues when they nag, beg, or demand us to do something in their favor. Moreover, reciprocity norms will likely ensure that both parties engaged in that conversation end up installing a CTA. In contrast to other health-compliant behaviors like wearing masks, app usage is not directly visible. Encouraging discussions about app usage might intensify normative social influence and hence increase the socially desirable installation of a CTA [8,11,30].

### Limitations

Several limitations of our study need to be considered when drawing conclusions. First, we used nonprobability sampling. However, as recommended in this case, we aimed for a broad sample, using different recruitment options and a diverse set of incentives for participation (money, course credit, personal feedback, and possibility for self-reflection). Moreover, our research question was neither the main focus of the questionnaire nor mentioned during recruitment, which decreased the risk of an association between self-selection and the target outcome [86]. Nevertheless, it is important to note that we used a web survey, and hence, participants might have a higher affinity for technology use, and that in respect to demographics, our sample is not representative of the general population in Switzerland and Germany. Therefore, we included demographic variables in our regression models to assess the association of concerns with app uptake independent of potential differences between demographic groups. The results should be carefully interpreted in consideration of the specific nature of our sample. Generalizability is also limited by the fact that our study only assessed the voluntary installation of two decentralized CTAs in two Western industrialized countries at a specific stage of the pandemic (after the first but before the second wave of COVID-19) [67,68] and in the context of moderate COVID-19–related policy responses [66]. It remains unclear whether the results hold for different app types and in different contexts [9].

Second, due to the nonexperimental nature of our data, it cannot provide evidence for causality. However, the experimental manipulation of health concerns during an ongoing pandemic would be ethically at least questionable. Moreover, the nonexperimental nature of our data collection facilitated the assessment of app installation instead of the intention for app uptake, avoiding potentially misleading conclusions. We strengthened our results by controlling for relevant variables that might be confounders. Our analyses showed that the association of self-focused concern with app uptake is not driven by the attitude toward COVID-19 or the government. When testing for reverse temporal order, we found that increased self-focused concern precedes app uptake and might hence be a driver of app installation. Nevertheless, the results on the predictors of app uptake only allow for the most cautious causal interpretation and should be considered only as hints toward such a relationship [87].

Third, we measured our predictor variables with single items. Particularly our item inquiring about participants’ concerns of unknowingly infecting others does not allow us to differentiate between concerns to infect close ties like partners or friends and concerns to infect colleagues at work or even complete strangers. While ample research shows that despite widespread criticism, single-item measures are not necessarily problematic in respect to their psychometric properties [88,89], future research would certainly benefit from using multi-item measures that allow for more differentiated insights.

### Conclusion

This study adds to the growing body of early research on CTAs. Hopefully, once the majority of the world’s population will have been vaccinated, the pandemic spread of COVID-19 will end and the use of CTAs will not be necessary anymore. However, diseases that quickly spread in the population have always been a threat and will likely continue to be [90]. If anything, increased mobility [91] and anthropogenic pressure on the environment [92] will make a new pandemic more likely. In case of a similar outbreak, reactivating privacy-preserving CTAs might help us to be better equipped to quickly contain new diseases, while reducing disruptions of normal life. Hence, a deeper understanding of individuals’ motivations to install CTAs is important not only right now but also in the future.

## Appendix

Multimedia Appendix 1 Logistic regression models predicting app uptake with concerns (T1).

Multimedia Appendix 2 Linear regression model predicting the change in “Concern self” with app uptake.

Multimedia Appendix 3 Linear regression model predicting the change in “Concern others” with app uptake.

Multimedia Appendix 4 Logistic regression model (M7) examining a curvilinear association between “Concern self” (T2) and app uptake.

Multimedia Appendix 5 Logistic regression model (M8) examining a curvilinear association between “Concern others” (T2) and app uptake.

## Abbreviations

AUC: area under the receiver operating characteristic curve
CTA: contact tracing app
OR: odds ratio
