# Supplementary material for: Self-Focused and Other-Focused Health Concerns as Predictors of the Uptake of Corona Contact Tracing Apps: Empirical Study
Source: J Med Internet Res. 2021 Aug 10;23(8):e29268. doi: 10.2196/29268 (PMC8360337; doi:10.2196/29268)
Supplement: Multimedia Appendix 1 [file jmir_v23i8e29268_app1.docx]

**Multimedia Appendix 1.** Logistic regression models predicting app uptake with concerns (T1).

|  |  |  |  |  |  | 95% CI_b_ | |  |
| --- | --- | --- | --- | --- | --- | --- | --- | --- |
|  |  |  | *b* | *SE*_a_ | *P* value | Lower | Upper | *OR*_c_ |
|  |  |  |  |  |  |  |  |  |
| **M4a** |  |  |  |  |  |  |  |  |
|  | Concern self T1 |  | 0.46 | 0.14 | <.001 | 0.20 | 0.74 | 1.59 |
| **M4b** |  |  |  |  |  |  |  |  |
|  | Concern others T1 |  | 0.16 | 0.10 | .12 | -0.04 | 0.37 | 1.18 |
| **M5** |  |  |  |  |  |  |  |  |
|  | Concern self T1 |  | 0.44 | 0.14 | <.001 | 0.17 | 0.72 | 1.55 |
|  | Concern others T1 |  | 0.09 | 0.11 | .42 | -0.12 | 0.30 | 1.10 |
| **M6** |  |  |  |  |  |  |  |  |
|  | Concern self T1 |  | 0.59 | 0.17 | <.001 | 0.27 | 0.93 | 1.81 |
|  | Concern others T1 |  | -0.13 | 0.15 | .37 | -0.42 | 0.15 | 0.88 |
|  | Satisfaction with government |  | 0.57 | 0.21 | <.01 | 0.17 | 0.99 | 1.76 |
|  | Not perceiving COVID-19 as health crisis |  | -0.45 | 0.15 | <.01 | -0.75 | -0.16 | 0.64 |
|  | Subsample Switzerland |  | 0.79 | 0.33 | .02 | 0.15 | 1.45 | 2.21 |
|  | Gender female |  | -0.52 | 0.41 | .20 | -1.34 | 0.26 | 0.60 |
|  | Age |  | -0.04 | 0.01 | <.01 | -0.06 | -0.01 | 0.97 |
|  | Education (ref.: Higher education) |  |  |  |  |  |  |  |
|  |  | Higher education entrance  qualification | -1.22 | 0.40 | <.01 | -2.03 | -0.43 | 0.30 |
|  |  | Vocational training | 0.35 | 0.52 | .49 | -0.63 | 1.41 | 1.42 |
|  |  | Lower to inter-mediate secondary education | 2.11 | 1.51 | .16 | -0.43 | 5.61 | 8.23 |
|  |  | Other/no degree | -0.30 | 1.65 | .86 | -3.83 | 3.26 | 0.74 |
|  | Political orientation (ref.: In the middle) |  |  |  |  |  |  |  |
|  |  | Extremely or somewhat left-wing | 0.30 | 0.39 | .44 | -0.47 | 1.05 | 1.35 |
|  |  | Extremely or somewhat right-wing | -0.94 | 0.61 | .12 | -2.16 | 0.24 | 0.39 |
|  |  | I don’t want to tell | -1.28 | 0.65 | .05 | -2.61 | -0.05 | 0.28 |

*_a_* _= Standard Error;_ *_b_* _= Confidence Interval;_ *_c_* _= Odds Ratio._
