# Supplementary material for: Self-Focused and Other-Focused Health Concerns as Predictors of the Uptake of Corona Contact Tracing Apps: Empirical Study
Source: J Med Internet Res. 2021 Aug 10;23(8):e29268. doi: 10.2196/29268 (PMC8360337; doi:10.2196/29268)
Supplement: Multimedia Appendix 2 [file jmir_v23i8e29268_app2.docx]

**Multimedia Appendix 2.** Linear regression model predicting the change in “Concern self” with app uptake.

|  |  |  |  |  | 95% CI_a_ | |
| --- | --- | --- | --- | --- | --- | --- |
|  |  | *b* | *t* | *P* value | Lower | Upper |
|  |  |  |  |  |  |  |
| Concern self T1 |  | 0.50 | 10.55 | <.001 | 0.41 | 0.59 |
| App-uptake |  | 0.14 | 1.44 | .15 | -0.05 | 0.34 |
| Satisfaction with government |  | 0.02 | 0.39 | .70 | -0.09 | 0.14 |
| Not perceiving COVID-19 as health crisis |  | -0.14 | -3.28 | <.01 | -0.23 | -0.06 |
| Subsample Switzerland |  | -0.02 | -0.21 | .84 | -0.21 | 0.17 |
| Gender female |  | -0.04 | -0.37 | .71 | -0.27 | 0.19 |
| Age |  | 0.00 | -0.68 | .50 | -0.01 | 0.00 |
| Education (ref.: Higher education) |  |  |  |  |  |  |
|  | Higher education entrance quali-fication | 0.05 | 0.42 | .68 | -0.19 | 0.29 |
|  | Vocational training | 0.11 | 0.75 | .45 | -0.17 | 0.39 |
|  | Lower to inter-mediate secondary education | 0.83 | 2.61 | <.01 | 0.21 | 1.46 |
|  | Other/no degree | 0.53 | 1.05 | .29 | -0.47 | 1.53 |
| Political orientation (ref.: In the middle) |  |  |  |  |  |  |
|  | Extremely or somewhat left-wing | -0.18 | -1.57 | .12 | -0.41 | 0.05 |
|  | Extremely or somewhat right-wing | -0.20 | -1.1 | .27 | -0.55 | 0.16 |
|  | I don’t want to tell | -0.60 | -3.31 | <.01 | -0.96 | -0.24 |

*_a_* _= Confidence Interval._
