# Supplementary material for: Self-Focused and Other-Focused Health Concerns as Predictors of the Uptake of Corona Contact Tracing Apps: Empirical Study
Source: J Med Internet Res. 2021 Aug 10;23(8):e29268. doi: 10.2196/29268 (PMC8360337; doi:10.2196/29268)
Supplement: Multimedia Appendix 3 [file jmir_v23i8e29268_app3.docx]

**Multimedia Appendix 3.** Linear regression model predicting the change in “Concern others” with app uptake.

|  |  |  |  |  | 95% CI_a_ | |
| --- | --- | --- | --- | --- | --- | --- |
|  |  | *b* | *t* | *P* value | Lower | Upper |
|  |  |  |  |  |  |  |
| Concern others T1 |  | 0.58 | 10.78 | <.001 | 0.47 | 0.68 |
| App-Uptake |  | -0.00 | -0.00 | >.99 | -0.25 | 0.25 |
| Satisfaction with government |  | -0.00 | -0.00 | >.99 | -0.15 | 0.15 |
| Not perceiving COVID-19 as health crisis |  | -0.13 | -2.33 | .02 | -0.25 | -0.02 |
| Subsample Switzerland |  | -0.22 | -1.83 | .07 | -0.46 | 0.02 |
| Gender female |  | -0.00 | -0.01 | >.99 | -0.30 | 0.30 |
| Age |  | -0.00 | -0.70 | .48 | -0.01 | 0.01 |
| Education (ref.: Higher education) |  |  |  |  |  |  |
|  | Higher education entrance quali-fication | 0.13 | 0.84 | .40 | -0.18 | 0.44 |
|  | Vocational training | -0.04 | -0.20 | .84 | -0.40 | 0.33 |
|  | Lower to inter-mediate secondary education | 0.65 | 1.59 | .11 | -0.16 | 1.46 |
|  | Other/no degree | -0.13 | -0.19 | .85 | -1.42 | 1.16 |
| Political orientation (ref.: In the middle) |  |  |  |  |  |  |
|  | Extremely or somewhat left-wing | 0.03 | 0.18 | .86 | -0.27 | 0.32 |
|  | Extremely or somewhat right-wing | 0.10 | 0.43 | .67 | -0.36 | 0.56 |
|  | I don’t want to tell | -0.16 | -0.68 | .50 | -0.62 | 0.30 |

*_a_* _= Confidence Interval._
