# Supplementary material for: Self-Focused and Other-Focused Health Concerns as Predictors of the Uptake of Corona Contact Tracing Apps: Empirical Study
Source: J Med Internet Res. 2021 Aug 10;23(8):e29268. doi: 10.2196/29268 (PMC8360337; doi:10.2196/29268)
Supplement: Multimedia Appendix 4 [file jmir_v23i8e29268_app4.docx]

**Multimedia Appendix 4.** Logistic regression model (M7) examining a curvilinear association between “Concern self” (T2) and app uptake.

|  |  |  |  |  | 95% CI_b_ | |  |
| --- | --- | --- | --- | --- | --- | --- | --- |
|  |  | *b* | *SE*_a_ | *P* value | Lower | Upper | *OR*_c_ |
|  |  |  |  |  |  |  |  |
| Concern self T2_d_ |  | 0.54 | 0.16 | <.001 | 0.22 | 0.86 | 1.71 |
| Concern self T2_d_ * Concern self T2_d_ |  | -0.09 | 0.12 | .44 | -0.32 | 0.15 | 0.91 |
| Concern others T2 |  | 0.01 | 0.11 | .90 | -0.21 | 0.24 | 1.01 |
| Satisfaction with government |  | 0.44 | 0.17 | <.01 | 0.12 | 0.78 | 1.56 |
| Not perceiving COVID-19 as health crisis |  | -0.34 | 0.13 | <.01 | -0.60 | -0.08 | 0.71 |
| Subsample Switzerland |  | 0.64 | 0.29 | .03 | 0.08 | 1.21 | 1.90 |
| Gender female |  | -0.54 | 0.33 | .10 | -1.19 | 0.09 | 0.59 |
| Age |  | -0.03 | 0.01 | <.01 | -0.04 | -0.01 | 0.98 |
| Education (ref.: Higher education) |  |  |  |  |  |  |  |
|  | Higher education entrance quali-fication | -1.13 | 0.36 | <.001 | -1.84 | -0.43 | 0.32 |
|  | Vocational training | 0.38 | 0.4 | .34 | -0.39 | 1.20 | 1.46 |
|  | Lower to inter-mediate secondary education | 0.13 | 0.85 | .88 | -1.49 | 1.93 | 1.13 |
|  | Other/no degree | -0.69 | 1.49 | .64 | -4.02 | 2.64 | 0.50 |
| Political orientation (ref.: In the middle) |  |  |  |  |  |  |  |
|  | Extremely or somewhat left-wing | 0.06 | 0.34 | .86 | -0.61 | 0.72 | 1.06 |
|  | Extremely or somewhat right-wing | -0.78 | 0.51 | .13 | -1.80 | 0.22 | 0.46 |
|  | I don’t want to tell | -0.55 | 0.52 | .29 | -1.58 | 0.47 | 0.58 |

*_a_* _= Standard Error;_ *_b_* _= Confidence Interval;_ *_c_* _= Odds Ratio; d = mean-centered._
