# Supplementary material for: Self-Focused and Other-Focused Health Concerns as Predictors of the Uptake of Corona Contact Tracing Apps: Empirical Study
Source: J Med Internet Res. 2021 Aug 10;23(8):e29268. doi: 10.2196/29268 (PMC8360337; doi:10.2196/29268)
Supplement: Multimedia Appendix 5 [file jmir_v23i8e29268_app5.docx]

**Multimedia Appendix 5.** Logistic regression model (M8) examining a curvilinear association between “Concern others” (T2) and app uptake.

|  |  |  |  |  | 95% CI_b_ | |  |
| --- | --- | --- | --- | --- | --- | --- | --- |
|  |  | *b* | *SE*_a_ | *P* value | Lower | Upper | *OR*_c_ |
|  |  |  |  |  |  |  |  |
| Concern self T2 |  | 0.48 | 0.16 | <.01 | 0.18 | 0.80 | 1.62 |
| Concern others T2_d_ |  | 0.02 | 0.11 | .87 | -0.21 | 0.24 | 1.02 |
| Concern others T2_d_*Concern others T2_d_ |  | -0.08 | 0.08 | .32 | -0.25 | 0.08 | 0.92 |
| Satisfaction with government |  | 0.45 | 0.17 | <.01 | 0.13 | 0.79 | 1.57 |
| Not perceiving COVID-19 as health crisis |  | -0.35 | 0.13 | <.01 | -0.61 | -0.10 | 0.70 |
| Subsample Switzerland |  | 0.64 | 0.29 | .03 | 0.08 | 1.21 | 1.90 |
| Gender female |  | -0.53 | 0.33 | .10 | -1.19 | 0.10 | 0.59 |
| Age |  | -0.02 | 0.01 | <.01 | -0.04 | -0.01 | 0.98 |
| Education (ref.: Higher education) |  |  |  |  |  |  |  |
|  | Higher education entrance quali-fication | -1.10 | 0.36 | <.01 | -1.82 | -0.41 | 0.33 |
|  | Vocational training | 0.42 | 0.41 | .30 | -0.36 | 1.25 | 1.53 |
|  | Lower to inter-mediate secondary education | 0.14 | 0.84 | .87 | -1.46 | 1.94 | 1.15 |
|  | Other/no degree | -0.68 | 1.48 | .64 | -4.00 | 2.64 | 0.50 |
| Political orientation (ref.: In the middle) |  |  |  |  |  |  |  |
|  | Extremely or somewhat left-wing | 0.05 | 0.34 | .89 | -0.63 | 0.70 | 1.05 |
|  | Extremely or somewhat right-wing | -0.79 | 0.51 | .12 | -1.81 | 0.21 | 0.45 |
|  | I don’t want to tell | -0.55 | 0.52 | .29 | -1.59 | 0.46 | 0.57 |

*_a_* _= Standard Error;_ *_b_* _= Confidence Interval;_ *_c_* _= Odds Ratio; d = mean-centered._
